# Severe aplastic anaemia after serial vaccinations for SARS‐CoV‐2, pneumococcus and seasonal influenza

**DOI:** 10.1002/jha2.443

**Published:** 2022-05-04

**Authors:** Xiao Wang, Dorottya Laczko, Gabriel C. Caponetti, Susan Rabatin, Daria V. Babushok

**Affiliations:** ^1^ Division of Hematology and Oncology Department of Medicine Hospital of the University of Pennsylvania Philadelphia Pennsylvania USA; ^2^ Department of Pathology and Laboratory Medicine Hospital of the University of Pennsylvania Philadelphia Pennsylvania USA; ^3^ Comprehensive Bone Marrow Failure Center Children's Hospital of Philadelphia Philadelphia Pennsylvania USA

**Keywords:** aplastic anaemia, case report, COVID‐19, immunization, paroxysmal nocturnal haemoglobinuria, SARS‐CoV‐2

## Abstract

We present a 67‐year‐old woman who developed progressive pancytopenia over 10 months, concomitant with administration of severe adult respiratory syndrome coronavirus‐2 (SARS‐CoV‐2), pneumococcal and influenza vaccines. She developed mild leukopenia ∼2 weeks after the SARS‐CoV‐2 mRNA vaccine sequence, with progressive symptoms after subsequent vaccines, eventually developing severe aplastic anaemia (SAA). While there have been several reports of vaccine‐related SAA, at time of submission, our case is the first reported to develop after the Moderna mRNA SARS‐CoV‐2 vaccine, as well as the first to document the gradual development of SAA over the course of many vaccine exposures. Physicians should be cognizant of vaccine‐associated SAA, considering current widespread coronavirus disease 2019 vaccination efforts.

1

Over the past 2 years, the coronavirus disease 2019 (COVID‐19) epidemic has impacted all aspects of health care. The advent of vaccines against severe adult respiratory syndrome coronavirus‐2 (SARS‐CoV‐2) has provided a vital tool in limiting the spread of disease.

With massive worldwide vaccination campaigns, rare haematologic side effects have been reported, including two published reports of severe aplastic anaemia (SAA) temporally following the Pfizer‐BioNTech mRNA vaccine for SARS‐CoV‐2 [1, 2]. Here, we report a third case of acquired SAA in this setting, developing in temporal association with serial immunizations, including the SARS‐CoV‐2 Moderna mRNA vaccine.

The patient is a 67‐year‐old woman with a reported history of Hashimoto's thyroiditis. Baseline complete blood count (CBC), performed 2 years before her index presentation, was normal, with white blood cell (WBC) count of 6.3 × 10^3^/μl, haemoglobin 13 g/dl, and platelet count 343 × 10^3^/μl (Figure 1). Approximately 9–10 months prior to her current presentation, the patient received the two‐dose Moderna SARS‐CoV‐2 mRNA vaccination series, which she tolerated without significant immediate side effects. Two weeks after her second vaccination, she was first noted to have mild leukopenia and anaemia on routine laboratory studies, with CBC showing WBC 3.5 × 10^3^/μl, haemoglobin 11.9 g/dl, platelet count 171 × 10^3^/μl, neutrophil count 1761 cells/μl, and a normal differential. Four weeks later (and 6 weeks total after her vaccination sequence), she developed further mild reductions in WBC and platelet count, as well as mild neutropenia, with repeat CBC showing WBC 3.3 × 10^3^/μl, haemoglobin 11.9 g/dl, and platelet count 146 × 10^3^/μl, with 1284 neutrophils/μl.

**FIGURE 1:**
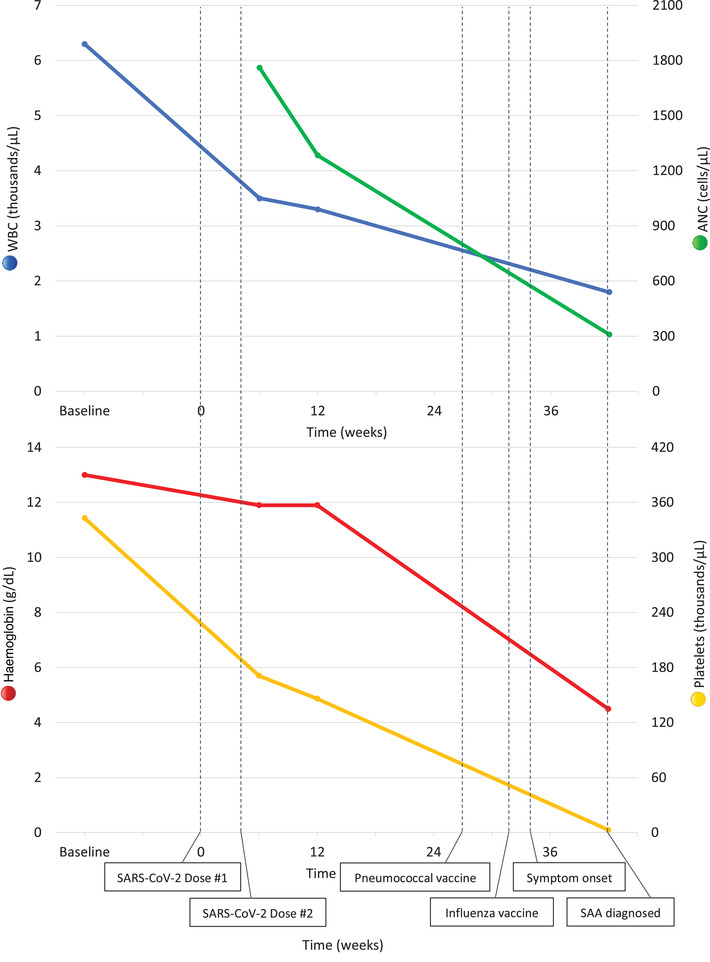
Approximate timeline of cytopenias and vaccinations. Baseline values represent laboratory values ∼2 years prior to her presentation. WBC = white blood cells, ANC = absolute neutrophil count

These persistent cytopenias prompted additional haematology evaluation, showing a weakly positive anti‐nuclear antibody (ANA; 1:80), normal thyroid function, negative hepatitis A/B viral serologies, and no nutritional deficiencies (vitamin B12, folate). She was given a presumed diagnosis of autoimmune leukopenia and was recommended to undergo biannual blood count monitoring. She continued to be asymptomatic during this time. Approximately 6 months after her SARS‐CoV‐2 vaccine series, she received a routine pneumococcal polyvalent booster (Pneumovax 23, Merck), followed by a seasonal influenza vaccine (Fluzone High Dose Quadruvalent, Sanofi Pasteur) about 5 weeks later, with no laboratory work obtained in the interim.

Two weeks after receiving her influenza vaccination, she began experiencing pulsatile tinnitus, leading to a normal otologic work‐up. Soon thereafter, approximately 1 month after her influenza vaccine, she began having visual disturbances, easy bruising, progressive dyspnea on exertion, and palpitations, despite having previously followed an active lifestyle; these prompted referrals to ophthalmology and cardiology. Eventually, her planned monitoring CBC was performed, revealing severe pancytopenia with WBC 1.8 × 10^3^/μl, haemoglobin 4.5 g/dl, and platelet 3 × 10^3^/μl, with 310 neutrophils/μl, reticulocyte count 0.8%, and immature platelet fraction 2.6%. She was admitted to the hospital for transfusions and further evaluation. An extensive investigation of causes of pancytopenia was unrevealing, including normal iron studies, vitamin B12, and folate levels; unremarkable inflammatory markers with negative ANA; and negative infectious work‐up for hepatitis B/C, human immunodeficiency virus, Epstein‐Barr virus, cytomegalovirus and parvovirus B‐19.

A bone marrow biopsy (Figure 2) revealed a hypocellular marrow (5%) with severe trilineage hypoplasia with a relative increase in lymphocytes, composed of CD3+ T‐cells and CD20+ B‐cells forming occasional clusters. CD34 immunostaining highlighted rare (<1%) blasts, with no morphologic or immunophenotypic evidence of myeloid or lymphoid neoplasms. Metaphase cytogenetics were normal, and single nucleotide polymorphism array analysis showed no evidence of a clonal process. Flow cytometry for paroxysmal nocturnal haemoglobinuria was also performed, revealing minor clones in monocyte (0.08%), neutrophil (0.65%), and red blood cell populations (0.02%).

**FIGURE 2:**
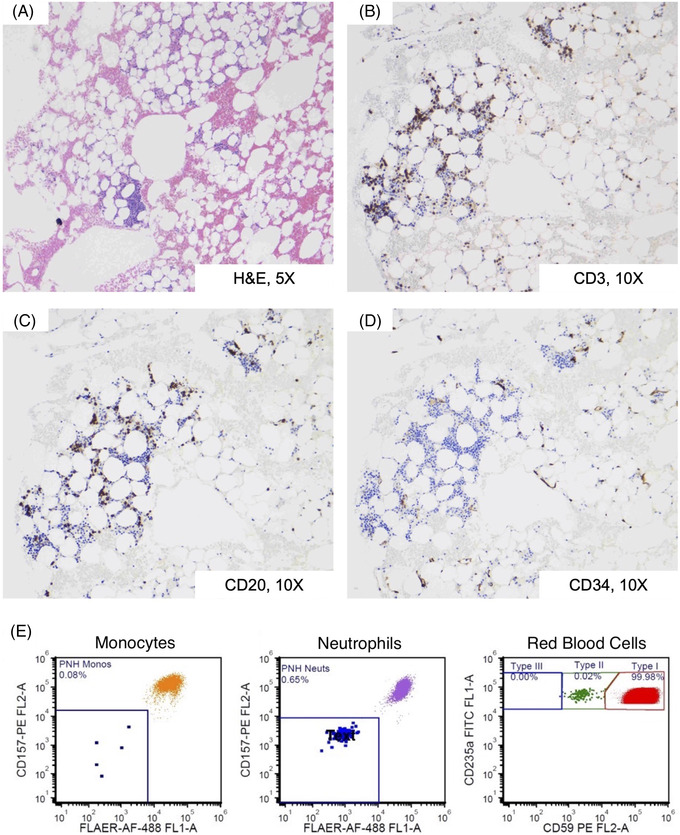
Bone marrow biopsy and paroxysmal nocturnal haemoglobinuria flow cytometry results. (A) Haematoxylin and eosin (H&E)‐stained sections at 10X magnification showing severely hypocellular marrow (5% cellularity), demonstrating haematopoietic trilineage hypoplasia. (B–C) Immunohistochemistry staining of bone marrow biopsy sections, shown at 10X magnification, demonstrates a relative increase in small lymphocytes composed of CD3 positive T‐cells (B) and admixed CD20‐positive B‐cells (C) forming occasional clusters. (D) Blasts were not increased, as demonstrated by immunohistochemical staining for CD34 (10X magnification) showing only rare myeloblasts (<1%). (E) A high sensitivity paroxysmal nocturnal haemoglobinuria (PNH) flow cytometry assay, performed on the patient's peripheral blood, demonstrated a minor GPI‐deficient PNH clones in red blood cells (0.02%) and neutrophils (0.65%), with rare PNH monocytes below the level of assay sensitivity (<0.3%), as shown by flow cytometry gates. Antibodies used: CD45, CD235a, CD15, CD64, CD59, CD157, FLAER

The patient was given a presumptive diagnosis of SAA and was treated with frontline immunosuppressive therapy with horse antithymocyte globulin, cyclosporine, and eltrombopag. She is currently continuing with cyclosporine, eltrombopag, and supportive transfusions, achieving transfusion independence and a near complete response 4 months after immunosuppressive therapy.

In summary, our patient developed SAA in temporal proximity to a series of vaccinations. Curiously, she was first noted to have mild persistent pancytopenia 4 weeks following the SARS‐CoV‐2 mRNA vaccination. While the initial cytopenias were mild and subclinical, she developed severe pancytopenia following further routine vaccinations administered several months later. Given the patient's history of autoimmunity with a history of hypothyroidism, we hypothesize that serial vaccines may have uncovered latent autoimmunity predisposing her to develop SAA, perhaps triggered non‐specifically by an immunization‐related general inflammatory response.

Multiple prior cases of new or relapsed SAA in close proximity to vaccinations have been reported, including vaccines for pneumococcus and seasonal influenza [3, 4], and more recently, two cases of SAA developing after Pfizer‐BioNTech vaccine for SARS‐CoV‐2 [1, 2]. A summary of cases found in the literature is found in Table 1. Our case is the first to document this more indolent presentation, with initial development of mild subclinical cytopenias after the SARS‐CoV‐2 vaccine sequence, followed by worsening severity with subsequent immunizations. Additionally of note, this is the first documented case of SAA temporally following the Moderna mRNA vaccine at the time of submission.

**TABLE 1:** List of case reports of vaccine‐related aplastic anemia. Adapted and updated from Ritz et al. [3]

Authors	Year published	Vaccine (manufacturer)	Time post‐vaccination
Viallard et al. [16]	2000	Recombinant hepatitis B (GenHevac, Pasteur Vaccines)	3 weeks
Ashok Shenoy et al. [17]	2001	Recombinant hepatitis B (Shantha Biologicals)	10 days
Hendry et al. [4]	2002	Influenza (Fluvirin: Medeva)	1 week
Shah et al. [18]	2004	Hepatitis B (unknown)	7 days
Shah et al. [18]	2004	Anthrax (unknown)	30 days
Angelini et al. [19]	2009	Varicella (Varivax III, Merck Frosst)	3 weeks
Donnini et al. [20]	2012	H1N1 influenza (Focetria)	2 days
Ritz et al. [3]	2020	Influenza (Flulaval Quadrivalent, ID Biomedical Corp. of Quebec); Pneumococcal (Pfizer)	1 week
Cecchi et al. [1]	2021	SARS‐CoV‐2 mRNA (Pfizer‐BioNTech)	1 month
Tabata et al. [2]	2021	SARS‐CoV‐2 mRNA (Pfizer‐BioNTech)	4 days
Present case	2022	SARS‐CoV‐2 mRNA (Moderna); Pneumococcus (Pneumovax 23, Merck); Influenza (Fluzone High Dose Quadruvalent, Sanofi Pasteur)	Gradual pancytopenia, first noted 2 weeks after SARS‐CoV‐2 vaccine, with progression and new symptoms 2 weeks after final vaccine

As with other reported cases of SAA occurring in proximity to vaccinations, proving the causality of these relationships, as well as deciphering their underlying immunologic mechanisms, remains challenging. Potential mechanisms of vaccine‐induced SAA include non‐specific immune stimulation caused by the inflammatory response to the vaccine and its adjuvant. Additionally, antigen‐specific expansion due to similarities between the administered vaccine and the presumed SAA autoantigen(s) is also possible. Indeed, we recently reported the case of SAA patient with massive clonal expansion of CD8+ T‐lymphocytes, with closely similarity of their T cell receptor complementarity‐determining region 3 (CDR3) to known influenza‐reactive CDR3 sequences [3]. In the current patient's case, the prolonged time‐course with multiple vaccines containing different antigens and adjuvants suggests that worsening SAA in this case was at least in part due to vaccine and/or adjuvant‐induced non‐specific immune stimulation.

The relationship between aplastic anaemia and vaccinations remains a complex one to navigate, especially in the context of the COVID‐19 pandemic. Certainly, while the incidences of autoimmune conditions developing in healthy individuals after SARS‐CoV‐2 vaccination may generate some degree of attention, such as concerns about myocarditis [5] and vaccine‐induced thrombotic thrombocytopenia [6], these remain rare, and the vast majority of side effects are mild and transient. Additionally, SARS‐CoV‐2 infection itself has been reported to produce similar and more severe complications, with several cases of SAA diagnosed in the setting of current or recent COVID‐19 infections [7, 8]. Patients with SAA are also at risk for developing severe COVID‐19 given their immunocompromised state and other comorbidities, although data on outcomes in this population are limited and mixed [7, 9–15]. In this context, we agree with local, national, and international organizations in recommending vaccines for healthy individuals.

In conclusion, the SARS‐CoV‐2 mRNA vaccines may be associated with the development of SAA in rare cases. Specifically with our patient, a history of autoimmunity and routine serial vaccinations may have contributed to the development of SAA. Physicians and other medical providers should be aware that vaccines, including SARS‐CoV‐2 mRNA vaccines, can potentially trigger the development of severe SAA or uncover milder, latent SAA in cases with no other identifiable causes. Routine blood count assessment is not required in healthy patients after routine vaccinations, as haematologic side‐effects are infrequent, and routine blood count surveillance is likely not cost‐effective. Nonetheless, our case highlights the importance of clinicians having a higher awareness of vaccine‐induced cytopenias, and blood count surveillance should be incorporated early in the diagnostic evaluation of persistent post‐vaccination symptoms. Additionally, our case suggests that patients with pre‐existing cytopenias should be monitored post‐vaccination, given the potential worsening of pre‐existing immune‐mediated haematologic conditions, such as SAA.

## FUNDING INFORMATION

The authors received no specific funding for this work.

## CONFLICT OF INTEREST

The authors declare they have no conflicts of interest.

## AUTHOR CONTRIBUTIONS

XW and DB reviewed the literature and wrote the manuscript. DL and GC performed haematopathology evaluation and provided photomicrographs. XW, SR and DB participated in the clinical care of the patient. All authors reviewed, edited and approved the manuscript.

## ETHICS STATEMENT

The patient's informed consent was obtained, and patient was enrolled into Penn‐CHOP Bone Marrow Failure Registry study, approved by the Institutional Review Boards of University of Pennsylvania and Children's Hospital of Philadelphia. All patient identifiers have been removed.

## Data Availability

Data sharing is not applicable to this article as no datasets were generated or analyzed during the current study.
